# Combining CNNs and Markov-like Models for Facial Landmark Detection with Spatial Consistency Estimates

**DOI:** 10.3390/jimaging9050104

**Published:** 2023-05-22

**Authors:** Ahmed Gdoura, Markus Degünther, Birgit Lorenz, Alexander Effland

**Affiliations:** 1 Department of Ophthalmology, Justus-Liebig-University Gießen, 35392 Gießen, Germany; ahmed.gdoura@augen.med.uni-giessen.de (A.G.); birgit.lorenz@augen.med.uni-giessen.de (B.L.); 2 Department of Mathematics, Natural Sciences and Data Processing, Technische Hochschule Mittelhessen, 61169 Friedberg, Germany; markus.deguenther@mnd.thm.de; 3 Department of Ophthalmology, University Hospital Bonn, 53127 Bonn, Germany; 4 Institute of Applied Mathematics, University of Bonn, 53115 Bonn, Germany

**Keywords:** facial landmark detection, convolutional neural networks, Markov random field

## Abstract

The accurate localization of facial landmarks is essential for several tasks, including face recognition, head pose estimation, facial region extraction, and emotion detection. Although the number of required landmarks is task-specific, models are typically trained on all available landmarks in the datasets, limiting efficiency. Furthermore, model performance is strongly influenced by scale-dependent local appearance information around landmarks and the global shape information generated by them. To account for this, we propose a lightweight hybrid model for facial landmark detection designed specifically for pupil region extraction. Our design combines a convolutional neural network (CNN) with a Markov random field (MRF)-like process trained on only 17 carefully selected landmarks. The advantage of our model is the ability to run different image scales on the same convolutional layers, resulting in a significant reduction in model size. In addition, we employ an approximation of the MRF that is run on a subset of landmarks to validate the spatial consistency of the generated shape. This validation process is performed against a learned conditional distribution, expressing the location of one landmark relative to its neighbor. Experimental results on popular facial landmark localization datasets such as 300 w, WFLW, and HELEN demonstrate the accuracy of our proposed model. Furthermore, our model achieves state-of-the-art performance on a well-defined robustness metric. In conclusion, the results demonstrate the ability of our lightweight model to filter out spatially inconsistent predictions, even with significantly fewer training landmarks.

## 1. Introduction

Facial landmark detection is commonly used as a preprocessing step for tasks such as face recognition, head pose estimation, emotion detection, and facial region extraction, with a specific interest in pupil region extraction (our case of study) for investigating inherited retinal diseases via pupillometry [1]. However, accurately recognizing predefined key points on the human face remains an unsolved problem, although significant progress has been made since its beginning in the 1990s by [2]. This is mainly due to different sources of variability, such as unconstrained illumination, extreme head poses, exaggerated expressions, occlusions, and out-of-plane configurations observed in the data. To address this issue, generative models, discriminative models, and hybrid models are typically employed, as described below. Facial landmark detection can be attempted using generative models, such as the active appearance models (AAM) introduced by [3], which were later adopted by [4], or using the active shape model (ASM) introduced by [5], which was later adopted by [6].

A second category of models used to address this problem involves exploiting discriminative models. This can be achieved through direct regression, as in [7], using heatmap-based CNNs, as proposed by [8], or through pixel-wise classification, as in [9]. Cascade shape regressions, another variant of discriminative modeling, can also substitute for discrete landmark estimation, where a set of regressors is learned to approximate the mapping between an initial shape and the ground truth, as proposed by [10,11]. it is worth stressing that recurrent neural networks can be exploited for facial landmark detection, as in [Recurrent neural network for facial landmark detection], but are usually outperformed by CNNs; however, RNN performance can be considerably enhanced via bifurication techniques; see [12,13,14,15,16].

Hybrid algorithms, as a third category, combine generative and discriminative approaches. For example, Ref. [17] exploits a CNN as a feature extractor to learn the input for the optimization process of an ASM to ultimately detect landmarks. In [10], the authors advocate face detection using the deformable parts model (PDM) combined with a cascade shape regression using multiscale histogram of oriented gradients (HOG) features (also utilized for registration techniques, as in [18]), by incorporating a local refinement for the least accurate landmarks. In [19], a CNN combined with a conditional random field (CRF) were jointly trained to capture the variations due to pose and deformation in order to generate a structured probabilistic prediction of landmark locations.

Finally, Ref. [20], which was originally designed for human pose estimation, conducted a joint training of a CNN and a Markov random field (MRF). The MRF validates the pairwise relationships between the already-estimated landmarks by the CNN based on the so-called learned conditional distribution of the location of one body part relative to another. In their work, they restricted the success of adopting these learned conditional distributions to the degree of their spatial constraint. More concretely, the set of potential positions of one landmark relative to its neighbor must be spatially consistent with its contextual limitations. As in their study case, spatial constraints can be majorly altered by back-facing poses, where, for instance, the left shoulder is no longer in the southwest direction of the nose. Moreover, extreme body poses are problematic, since they could lead to overlaps between landmarks.

In our study, we utilized learned conditional distributions to confirm the spatial consistency of pre-estimated landmark locations, motivated by three reasons. Firstly, unlike human pose estimation, back-facing images in facial landmark datasets are considered invalid and excluded. Secondly, extreme head poses are expected to result in fewer spatial inconsistencies compared to extreme body poses. Lastly, upside-down frames are rare in the training datasets we employed, and therefore not relevant to our inference case. This confirmation process is referred to as the spatial model (SpatialModel). The initial landmarks estimation is achieved via a multiscale heatmap-based CNN, referred to as the landmark detector (LandmarkDetector). Our aim in combining these elements is to enhance the ability to handle both the local appearance information around landmarks and the global shape information they generate.

The next challenge that needs to be addressed is complexity. As we have seen, various strategies can be employed to tackle facial landmark detection; however, the underlying task is often overlooked, and models are constructed and trained without explicitly considering it. In our opinion, designing a landmark estimator based on the underlying task could drastically alleviate the model complexity.

Therefore, the computational complexity was reduced by taking three measures, as explained above:

(i) The model was trained exclusively on 17 carefully selected landmarks preserving the global shape of the face. Additionally, these landmarks placed more emphasis on the pupil region, with 12 landmarks specifically positioned on the eyebrows and eyes, as demonstrated in Figure 3.
(ii) Different image scales were run through the same layers instead of assigning a new convolution layer for each scale.
(iii) SpatialModel runs only a subset of the available landmarks to validate their spatial consistency, instead of running the full set of them.

Our combination of LandmarkDetector and SpatialModel serves as a means of eliminating false positives, thereby increasing the certainty of LandmarkDetector with regard to landmark locations. This is explained in detail in Section 4.3.1. To ensure that the landmarks’ locations and their relative pose consistency are learned jointly, we introduce a customized loss function that directly influences the CNN, as presented in Section 3.3. In the following section, we explore the significance of CNN models, MRF-based models, and their combinations for facial landmark detection. We introduce the model components and their combination via a customized loss function in Section 3. Finally, in Section 4, we present our model’s performance metrics, and show that we achieve a normalized mean error (NME) of 3.3% and 4.1% for the 300w and WFLW datasets, respectively. In addition to high accuracy, we achieve a state-of-the-art robustness performance, as evidenced by a robustness metric by investigating NME for 90% of the predictions, and we report 4.0% for the 300 w dataset.

## 2. Related Work

A successful facial landmark detection needs to address two main challenges: local context distinguishability, which should be obtained from the surrounding area of the subject landmark; and global compatibility, which should adhere to contextual spatial constraints. To solve both problems, we propose a hybrid model that combines a CNN with a generative model.

Although CNNs are effective in distinguishing local features due to the small size of their low-order kernels compared to the input image, high-order kernels are not efficient in learning global context due to the low resolution of the receptive fields they process.

Yue et al. (2015) in [21] demonstrated that feature quality gradually increases from low to intermediary layers, then drops again when progressing towards the last layers.

In contrast, statistical models can effectively learn the higher-level constraints of landmark configurations, which enables them to enforce global spatial consistency on a given set of pre-estimated landmarks. To determine the characteristics of our model, we briefly investigate a range of CNN-based approaches for facial landmark detection. We then introduce our choice of the generative model.

### 2.1. CNN Characteristics

In this section, we provide a brief overview of CNN-based methods that have been used to determine the characteristics that our proposed network should possess. Early attempts by [22], and later by [23], involved performing cascade regression of facial landmark locations using a multilevel convolutional network model. In their work, a multistage, multi-input CNN was simultaneously executed on different subregions of a face-bounding box, also known as patches. As the patches progressed to the next convolution stage, they became narrower around the target landmark, resulting in progressively refined estimations.

However, this coarse-to-fine prediction approach heavily relies on the accuracy of the initial face detector and the cropping process around the subject set of landmarks, which can lead to inaccurate results if the input image presents moderate-to-extreme head poses or an out-of-frame part of the face. Hence, our proposed model does not rely on initial face detection procedures to perform its task.

Our model’s next desirable feature is flexibility towards ablation, which refers to the ability to handle inputs with missing landmarks. To better understand this feature and its impact on the overall performance of landmark detection, we begin by exploring direct regression-based CNNs. These models attempt to learn direct mapping from the input image space to the landmark coordinates. However, in papers such as [22,24], the output dimensionality of the network is fixed, which limits their ability to handle ablated frames that may contain a variable number of landmarks. Additionally, ablated frames cannot be integrated into the training phase of these models because the optimization criteria require fixed-length ground-truth coordinates. Furthermore, highly nonlinear mappings are prone to poor performance compared to heatmap-based mapping, where the complexity is reduced due to the proportional similarity between input and output spaces. In fact, the performance of direct regression-based methods, such as the one proposed in [25], was evaluated in Table 9 of [26], where it was outperformed by all mentioned heatmap-based methods.

Furthermore, it is common for direct mapping CNNs to terminate with a flattening layer, followed by a series of fully connected layers to downsample the data to meet the landmark dimensionality in their 2D coordinate space, as in [22]. However, recent works tend to substitute such heterogeneous networks with fully convolutional networks (FCNs), where special convolution layers are adopted to achieve end-to-end convolutional learning and inference, as extensively argued in [27]. The importance of adopting FCNs lies in their ability to preserve the spatial structure of the input signal, opposite to flattening layers, resulting in less complex mapping. Moreover, the 1D flattening of the signal necessitates more parameters, making them more prone to overfitting. Finally, FCNs enable arbitrary-sized input images, which provides another type of flexibility. For the aforementioned reasons, we conclude that our chased CNN must belong to the FCN category.

It is worth noting that [28] was able to overcome the complexity of the learned mapping by using a CNN-FC network for facial landmark detection. This was achieved by sequentially reducing the input space via splitting regions around the sought landmarks. However, this approach expects a fixed number of landmarks, and therefore cannot efficiently cope with ablation.

The following is a quick overview of FCN, which is the type of CNN we have chosen to use.

In 2015, Ref. [29] successfully demonstrated the efficiency of FCN by transforming the classification network into a classification and segmentation task, which was interpreted from an output heatmap. Since then, various approaches have derived different varieties of FCN, which can be split into two main categories: encoder–decoder (such as U-Net, introduced by [30], and Hourglass networks, introduced by [31]) and decoder-only networks, which are also known as heatmap regression networks, as introduced by [32]. Encoder–decoder FCNs, originally introduced by [29], are usually adopted to generate heatmaps with the same size as the input, which are generally used for pixelwise classification (PWC). However, for our purposes, we have chosen the decoder-only network, which produces lower-size heatmaps that we interpret as a probabilistic indicator of the locations of the facial landmarks. Therefore, heatmap regression is our adopted FCN type among CNN-based regressions. Compared to direct regression, heatmap regression commonly requires even fewer trainable parameters, since the target mapping is usually less complex due to the same input and output image dimensionality. In addition, unlike direct regression, heatmap regression can naturally handle amputated frames, which is a source of variability that can be expected in real-life data and free-head pose tracking experiments due to their flexibility toward input and output dimensionality.

### 2.2. Postprocessing by Generative Models

Generative models have been found to be useful for deep learning, as shown by [33]. They are employed to refine the initial estimates of the CNN and ensure the global consistency of their final output. Spatial consistency constraints are modeled by incorporating information about the interconnectivity of the facial landmarks in the learning process. Graphical models, including MRF or CRF, are popular approaches for integrating interactions between landmarks. These models capture geometric properties such as shape, spatial relationships, and connectivity among landmarks. Specifically, they estimate conditional probabilities of one landmark given the rest of the predicted landmarks, and the degree of compliance of the estimated probability with the implicit conditional probabilities from the training data is a measure of the spatial consistency. However, integrating graph-based models with CNN to build an end-to-end system for learning and inference has been challenging, and the implementation details must be carefully studied. One issue is deciding whether to adopt an approximation, as in [19], or to integrate the exact formulation of the probabilistic graphical model, as in [20].

In this work, we adopt an MRF-like process to maintain a low complexity level of this postprocessing step. We propose a simplified approach for conditional probability learning, leveraging the Gaussian mixture model tool. Furthermore, we suggest our approach for integrating the statistical model into the training framework via a customized loss function.

## 3. Materials and Methods

Our proposed method for facial landmark detection exhibits two major components: the landmark detector (see Section 3.1), which estimates the location of landmarks based on heatmaps that represent the probability of their occurrence at a specific position; and the spatial model (see Section 3.2), which verifies the pose consistency of a landmark relative to the other ones in accordance with a Markov random field-like graph in the postprocessing step. Finally, we introduce the loss function, which enables simultaneous training of both components in Section 3.3. As studied by [34], a useful feature map is a representation that includes the following:

(i) High-level features generated from a sufficiently deep network to encode high-level object knowledge;
(ii) Fine spatial details around the object in order to learn its discriminativeness;
(iii) An explicit internal representation of entities and their relationship to associate components with one another.

Our hybrid model should therefore handle the aforementioned requirement through its components. More concretely, the landmark detector is designed to handle (i) and (ii), whereas the spatial model is responsible for (iii).

### 3.1. Landmark Detector

**Figure 1:**
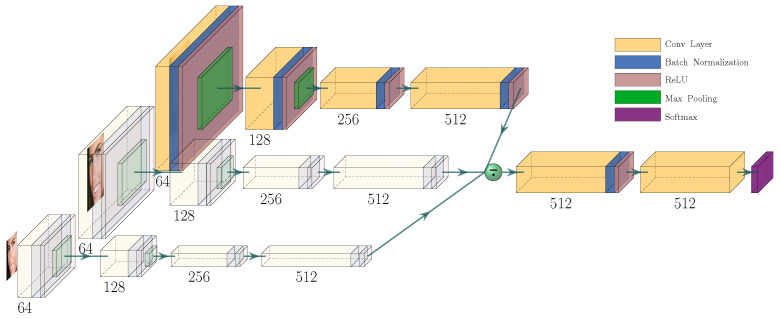
The proposed CNN: transparent effect means no new layer; the average is computed at the green circle level (best viewed in color).

The CNN-based landmark detector, as presented in Figure 1, is designed to generate heatmaps (one for each predefined landmark) reflecting the probability distribution of a specific landmark being located at a specific position.

Our depicted network exhibits two subparts (S1) and (S2):

(S1) Each image is processed on three different scales by four consecutive convolutional blocks, which essentially extract low-order features.
(S2) Subsequently, the average of the results of the previous subpart represents the input for the remaining convolution layers, which extract the higher-order features to ultimately generate LandmarkDetector’s output.

This architecture, via its two subparts, is designed to equip our LandmarkDetector with discriminative local features simultaneously with enough high-level knowledge, as explained in Section 3.1.2. Moreover, the distributed design of (S1) tends to explicitly learn scale invariance to efficiently deal with variations in the sizes of the objects, as explained in the forthcoming subsection.

#### 3.1.1. Scale Variance Handling

According to [35], scale invariance cannot be considered an intrinsic feature of CNNs, and is strongly affected by image resolution. Therefore, if we want to pursue this feature, we must explicitly teach our model to learn scale-variant information. This can be achieved by exposing the model to this type of information at different scales.

The first and most straightforward method to achieve this is by augmenting the training data using scale jittering. This technique partially and arbitrarily zooms images within a predefined scale range. While this approach can be seen as a way to expose the model to more data and reduce overfitting, Ref. [36] showed that it leads to the model requiring more scale-variant versions of the same learned feature instead of learning a scale-invariant feature. This increases the model size, especially when many scale levels are introduced, and results in overfitting.

The second approach is to train separate CNNs at different scales and average their estimations for the final output. However, this approach suffers from redundancy, particularly for scale-invariant or high-level features, and cannot scale up if a large scale range is expected.

To overcome these limitations, we designed (S1) to systematically incorporate each data point at three different scales through the same layers. This technique aims to push the first convolutional blocks of the network to build scale-invariant representations out of scale-variant features. By exposing our model to each data point at different scales, we can avoid introducing new convolutional layers and build more efficient and effective scale-invariant representations.

#### 3.1.2. Low-/High-order Feature Compromise

While low-order features have a limited interpretation of objects, high-order features offer a more generalized understanding due to their larger receptive field. However, increasing the receptive field by using size reduction layers in the CNN leads to deteriorated spatial resolution due to multiple subsampling steps, resulting in failure to provide local context variation. This is particularly challenging for very deep networks, as they struggle with local distinguishability. To address this issue, we widen our network inspired by [37], to promote the depth of the learned feature and distinction of the local context simultaneously.

To achieve a fine-spatial, sufficiently deep, multi-scale handling feature map, we adopt a CNN with a distributed architecture that runs the Gaussian pyramid of every image, as illustrated in Figure 1. We note that the shallow scale-distributed architecture of subpart (S1) of our LandmarkDetector preserves spatial affinity, enabling us to maintain local discriminativeness. We equip the second subpart (S2) of our network with high-dimensional convolutions, essential for learning high-order features.

Finally, our network generates heatmaps that present landmark-specific unary distributions, indicating the probability of the presence of a subject landmark at each pixel’s coordinates.

We demonstrate in the following section, Section 3.2, how these heatmaps are fed to SpatialModel to perform a spatial consistency check of each detected landmark relative to a predefined set of other landmarks.

### 3.2. Spatial Model

Given the initial landmark’s unary distributions estimated by LandmarkDetector, and revealing their locations, we postprocess them via an MRF-like process to validate their relative spatial consistency.

For this purpose, we treat every landmark *i* via a landmark-specific graph model $G_{i}$, built out over a predefined set of its neighbors. Thereafter, we run the MRF-like process over the vertices of $G_{i}$ to catch spatially correlated features characterizing their mutual influences. In this process, we exploit the learned conditional distributions from the training data, as presented by [38] and detailed in the next section:

#### 3.2.1. Learned Conditional Distribution

The learned conditional distribution for a pair of landmarks (*i*, $cond_{i}$), noted $p_{i|cond_{i}}$, is determined offline before starting any model training procedures. For each image, we translate the landmark *i* with the same amount that would shift its conditional landmark $cond_{i}$, located at $\left(u_{cond_{i}},v_{cond_{i}}\right)$ to the frame center. We quantify this translation amount by

(1) $$T_{i|cond_{i}}=\mathrm{center}\;\mathrm{of}\;\mathrm{frame}-\left(u_{cond_{i}},v_{cond_{i}}\right)$$

After landmark coordinates are transformed, conditional probabilities were built for every $i|cond_{i}$ combination via Gaussian mixture model (GMM). Usually used as a classification technique, as in [39], we exploited GMM to fit the data points to a mixture of a finite number of Gaussian distributions, which is also called the order, with unknown parameters. Order determination was achieved via an exhaustive investigation of the fitting performance based on predefined scores. For each combination $i|cond_{i}$, the GMM order was established as the mean of the minimum AIC and BIC scores. Typically, the GMM order ranged from 7 to 9.

**Figure 2:**
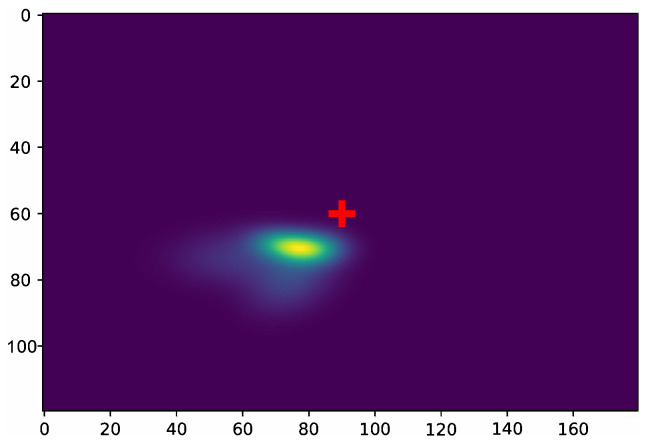
*p_lmouth|nose_*: The left mouth extremity spatial distribution when nose landmark occupies the heatmap center (+) (Best viewed in color).

Note that the resolution of the learned conditional probabilities was twice the resolution of the estimated heatmaps. Figure 2 illustrates the conditional probability $p_{lmouth|nose}$, revealing the locations of occurrence of the left extremity of the mouth when the nose tip was located on the frame center $center_{120\times 180}$.

In [38], the pre-estimated heatmap of a landmark was filtered by the learned conditional distributions of its direct neighbor according to an approach analogous to the sum–product belief propagation algorithm introduced by [40]. In our case, we explore a broader neighborhood space for validating the spatial consistency of the subject landmark.

#### 3.2.2. Neighborhood Space Definition

To define neighborhood systems for the MRF as a neighborhood-based graph model, we proceed as follows: Let S={1,2,…,n} be the set of *n* landmarks and i∈S a specific landmark. Then, the associated local neighborhood $N_{i}\left(r\right)$ for the landmark *i* given a radius r>0 reads as

(2) $$N_{i}\left(r\right)=\left\{i^{\prime }\in S:dist\left(i,i^{\prime }\right)\leq r,i^{\prime }\neq i\right\},$$

where $dist(i,i^{\prime })$ is the Euclidean distance between *i* and $i^{\prime }$. In addition to the local neighborhood, we consider the fixed set of global reference landmarks $N_{g}\subset S$, which encompass distinct particularly conspicuous facial landmarks.

**Figure 3:**
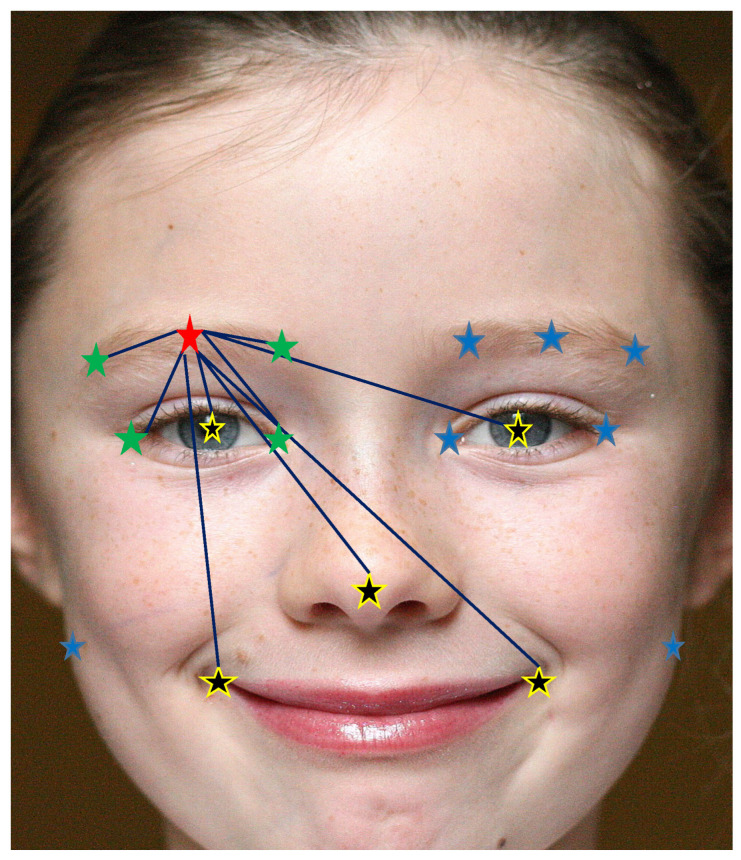
MRF Graph Gi for a specific landmark (red) linked to its local neighborhood Ni(r) (green) and to the global one Ng (yellow); the rest of the landmarks are discarded for this case (blue) (best viewed in color).

Finally, our customized neighborhood $N_{u}\left(i\right)$ is the union of $N_{i}\left(r\right)$ with $N_{g}$, such that $N_{u}\left(i\right)=N_{i}\left(r\right)\cup \;N_{g}$ as detailed in Figure 3 (Image by Vincent Angler (CC BY-2.0), https://commons.wikimedia.org/wiki/File:Croydon_facelift_2012.jpg (accessed on 1 May 2023).

It is worth noting that the choice of conditional landmarks obeys the following rules:

- $N_{i}\left(r\right)$ presents the local consistency challenge that prioritizes the nearest neighbors over farther ones and provides an image of the local state around the subject landmark.
- $N_{g}$ presents the global structure of the human face that prioritizes some landmarks, which we call central landmarks, over others. $N_{g}$ roughly indicates the smallest set that describes most of the face structure.

The landmark-specific neighborhood sets now present our structure elements of the graph model, over which we run our MRF-like process.

#### 3.2.3. Landmark-Specific Graph Definition

Every landmark *i* is treated in SpatialModel via a graph $G_{i}$ built over the subject landmark and its neighbor set $N_{u}\left(i\right)$, and executed by the MRF-like process.

The vertices of each landmark-specific graph are confounded with the set of landmarks $\left\{i\cup \;N_{u}\left(i\right)\right\}$. The edges of the graphs, however, are restricted in order to link the subject landmark vertex to its neighbors’ vertices and discard every interneighbor relationship. Note that this measure drastically alleviates SpatialModel’s complexity, as it runs an iterative process proportionally dependent on the degree of the treated graph (number of edges). Based on our graphs’ definitions, fully connected subgraphs, also called cliques, over which the MRF-like process should be executed, are restricted to bivertex graphs (i,j) where $j\in N_{u}\left(i\right)$.

Now that we have defined our landmark-specific graphs $G_{i}$s over the landmark-specific neighborhood $N_{u}\left(i\right)$, in the following, we will discover the adopted process and its approximation for inferring the marginal probabilities of landmarks’ locations given the locations of a subset of other landmarks.

#### 3.2.4. SpatialModel Implementation

For a landmark i∈S, we denote by $x_{i}=\left(u_{i},v_{i}\right)\in R^{2}$ the random variable associated with its spatial coordinates. We also denote by $p(x_{i})$ the unary marginal probability, indicating that a landmark *i* is located at the site $x_{i}$. For simplicity, we write $p_{i}$ to indicate $p(x_{i})$, and ${\hat{p}}_{i}$ is its approximation by SpatialModel. Ref. [20] adopted the following potential-like function, where the unary marginal probability of a landmark *i* is inferred given the position of all other landmarks:

(3) $${\hat{p}}_{i}=\frac{1}{Z}\prod_{j\in N_{u}\left(i\right)}p_{i|j}*p_{j}+b_{j\to i}$$

where $p_{i|j}$ is the learned conditional prior of the pairs of landmarks (i,j), $b_{j\to i}$ is a bias term used to describe the background probability for the message passing from a landmark *j* to *i*, ∗ presents the convolution operation, and *Z* is a normalization function that will be later discarded in the model approximation presented in (4). Similarly to [20], we adopt Equation (3) and run it over every clique (i,j)$/j\in N_{u}\left(i\right)$ in $G_{i}$.

The SpatialModel task can be summarized as an incremental filtering process of LandmarkDetector’s assumption against its predefined neighbors to consolidate or inhibit this assumption, as detailed below.

First, we define lsp as the log of the Softplus equation, where $\mathrm{Softplus}\left(x,\beta \right)=\frac{1}{\beta }\ln\left(1+e^{\beta x}\right)$. lsp is applied to $p_{i}$ to convert it into an initial marginal energy $me_{i}=lsp\left(p_{i}+\varepsilon \right)$, such that ε>0.

Then, for every predefined conditional landmark (also called neighbor) in $N_{u}\left(i\right)$, we convolve $p_{i|cond_{i}}$ with $p_{cond_{i}}$ after being Softplus-transformed, and before being log-transformed. The acquired quantity is iteratively added to the initial quantity $me_{i}$. The final quantity is exponentially transformed to return back from the initially applied log-transformed space.

The described process can be summarized by the following equation:

(4) $${\hat{p}}_{i}=\exp\left(me_{i}+\sum_{cond_{i}\in N_{u}\left(i\right)}{\ln[\mathrm{Softplus}(p}_{i|cond_{i}})Softplus\left(p_{cond_{i}}\right)+bias+\varepsilon ]\right)$$

Note that (4) does not quantify a probability anymore due to the bench of approximations that were applied to (3). For simplicity, we rather preserve the ${\hat{p}}_{i}$ notation to indicate SpatialModel’s output. The outer multiplication in (3) is substituted with the log-space addition, which controls the scale of the resulting quantities, and hence improves the numerical stability. In addition, the Softplus function is introduced to maintain a strictly greater-than-zero convolution output, avoiding numerical issues for input quantities of the log stage. The 2D convolution in (4) can be perceived as an incremental update of a landmark’s position by its neighbors. The update’s level is relative to the degree of agreement between the intensity at the estimated landmark location in $p_{i}$ to their corresponding $p_{i|j}$. In other words, SpatialModel searches for the best landmark’s location that agrees simultaneously with LandmarkDetector’s estimation, as well as with its neighbors based on their relative conditional probability $p_{i|j}$.

For a better adaptation to the LandmarkDetector model,  SpatialModel was implemented as convolutionally as possible. In fact, we leveraged the grouped convolution function provided by PyTorch to simultaneously convolve each learned conditional distribution to their corresponding estimated one.

Similarly to LandmarkDetector, it is worth stressing that SpatialModel also produces landmark-specific heatmaps, revealing their locations. However, we only introduced the coordinate of their maximum to the learning process combined with LandmarkDetector’s output to build a heterogeneous loss function, introduced in Section 3.3. Therefore, the heatmaps positions and their spatial validity were simultaneously evaluated after being provided by LandmarkDetector’s predicted heatmaps and the Cartesian coordinates of SpatialModel, respectively.

### 3.3. Loss Function

As proposed earlier, the adopted loss function is a combination of two terms. The first one evaluates the accuracy of LandmarkDetector in estimating the positions of the facial landmarks. The second term penalizes the detected landmarks’ deviation from the anatomical constraints learned by SpatialModel. Therefore, the combination of LandmarkDetector and SpatialModel is evident at the loss function level.

To evaluate the accuracy of LandmarkDetector, we employed the adaptive wing loss function proposed by [41]. While the mean-square error (MSE) is commonly used to compare two heatmaps, it fails to distinguish between foreground and background pixels. In fact, MSE, as a distance-based loss function, will produce low error values whenever the mass background pixels, i.e., pixels far from the landmarks, are satisfactorily estimated, and therefore tends to dominate the loss value and results in fuzzy heatmaps around the ground-truth locations. Our adopted function, however, is specifically designed to handle this issue. It gives more importance to the foreground pixels’ error in the early stages of training, where the overall error is high, then rapidly decreases when the model is close to convergence, i.e., the error is within the predefined tolerances. Meanwhile, the influence of the background pixels is linearly proportional to the overall error. The AWing loss between two pixel values, *y* and $\hat{y}$, is defined as follows:

(5) $$\mathrm{AWing}\left(y,\hat{y}\right)=\left\{\begin{array}{cc} \omega \ln(1+{\left|y-\hat{y}\right|}^{\alpha -y}), & \mathrm{if}|y-\hat{y}|<\theta \\ A|y-\hat{y}|-C, & \mathrm{otherwise} \end{array}\right.$$

where *y* and $\hat{y}$ are the pixel values of the ground-truth heatmap and the predicted heatmap, respectively. α, ω, ϵ, and θ are positive values, to which we assign the values suggested in the original paper, i.e., 2.1, 14, 1, 0.5, respectively.

$$\begin{array}{cc} A & =\omega \frac{1}{1+{\frac{\theta }{\varepsilon }}^{\left(\alpha -y\right)}}\left(\alpha -y\right){\left(\frac{\theta }{\varepsilon }\right)}^{\left(\alpha -y-1\right)}\frac{1}{\varepsilon }\\ C & =\theta A-\omega \ln\left(1+{\left(\frac{\theta }{\varepsilon }\right)}^{\alpha -y}\right) \end{array}$$

The previous parameters were carefully defined to make the loss function continuous and smooth at $|y-\hat{y}|=\theta$. Note that the exponential term α−y adapts the shape of the loss function to *y* and smooths the function a 0.

Without loss of generality, we redefine the AWing loss between two heatmaps, each containing *N* pixels, as the mean of all their pixel errors, as follows:

(6) $$\mathrm{AWing}\left(hm_{1},hm_{2}\right)=\frac{1}{N}\sum_{\left(y_{1},y_{2}\right)\in hm_{1}\times hm_{2}}\mathrm{AWing}\left(y_{1},y_{2}\right)$$

Coming to the second part of the loss function, we run the mean square error (MSE) between the SpatialModel prediction and the ground-truth landmark coordinates. Even though, as argued above, MSE is by no means the optimal loss function for heatmap regression, it is worth stressing that the calculated error reveals the consistency metric rather than accuracy. In this case, the gradient linearity of MSE enables treating inconsistencies according to their magnitude. While this linearity is disapproved for accuracy evaluation as it leads to convergence, even when many pixels still have small errors, small inconsistencies, on the other hand, should not cause the same effect on the overall performance of the model. Based on the validation error of the LandmarkDetector model vs. the mentioned combination, one could claim that enforcing facial parts constraints incites the model to enhance its prediction. The aim behind SpatialModel integration is not about seeking higher accuracy (which should be guaranteed by the CNN alone), but rather, about detecting and penalizing false positives. The joint learning of the consistency and the accuracy is translated by the following heterogeneous loss function, which combines the provided metric of SpatialModel with the LandmarkDetector estimations.

The final loss quantity of a predicted landmark and its ground truth is summarized as follows:

(7) $$\mathrm{Loss}=\left(1-\beta \right)\mathrm{AWing}\left(hm,hm_{ld}\right)+\beta {\left|x-x_{sm}\right|}_{2}^{2}$$

where β is set to 0.1. The previous equation presents a weighted sum between the AWing’s output, comparing the ground-truth heatmap hm to the predicted one by LandmarkDetector, $hm_{ld}$, on one side, and the square of the $L_{2}$ distance between the ground-truth landmark coordinates *x* and the predicted coordinates by SpatialModel, $x_{sm}$, on the other side.

## 4. Results and Analysis

In this section, we present the training experiments and their resulting outcomes on the adopted datasets. Two different experiments were carried out with the aim of investigating the impact of the model’s two components (LandmarkDetector and SpatialModel) on the overall accuracy in three different datasets. We started by training only the LandmarkDetector; then, we launched a second training experiment combining both models’ components. Our data enhancement procedure is extensively addressed in Section 4.1. In Section 4.2, we present the training details in terms of the adopted hyperparameters such as the input size, the batch size, the learning rate, the number of epochs, and the data augmentation details. Finally, the model’s performance was qualitatively tested, in addition to a quantitative evaluation based on well-defined metrics, as can be consulted in Section 4.3.

### 4.1. Data Enhancement

Common facial landmark databases provide a significant number of annotations (e.g., 98 landmarks for WFLW; 68 for 300w and LFPW), which is generally useful for tasks such as facial recognition or facial expression analysis. On the other hand, tasks such as facial parts localization (our study case) or head pose estimation would obviously require fewer annotations. Driven by the overall complexity alleviation of the process, we decide to train our model with a limited yet sufficient amount of landmarks, annotating well-defined points, and preserving the global facial shape. In other words, the number of landmarks should be defined by a lower limit, below which the global facial outline will be drastically affected. The significance of this lower bound emerges from the “Sufficient Landmark Density” concept, where studies analyze the dependency between the optimization quality and the number of landmarks constituting an active shape mode. In fact, Refs. [42,43] showed that ASM must be modeled with a sufficient landmark density to reach some fitting accuracy level.

Our initial thoughts were to simply select a subset from the provided landmarks that fulfilled our needs.

**Figure 4:**
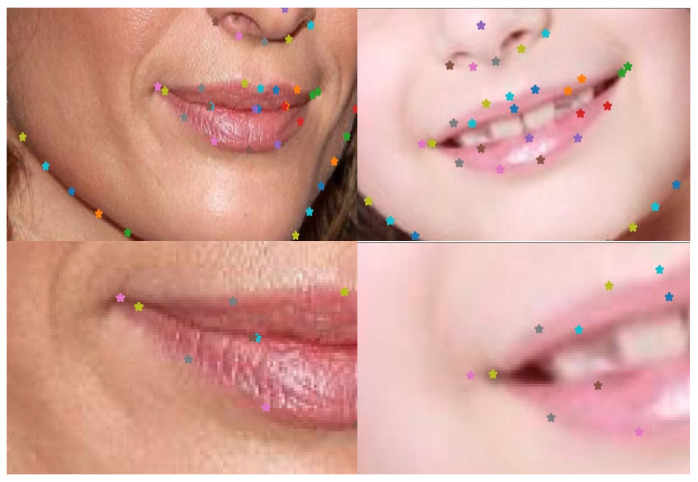
Examples of inconsistent labeling, where the same color should present the same facial landmark. However, the mouth extremity is presented in pink ((**left**) image) and in yellow ((**right**) image). A zoom of the region is presented in the second row (best viewed in color).

However, after investigating the exploited datasets, we found out that the landmarks form contours around facial parts, rather than annotating well-defined points. In Figure 4 (Image from the HELEN dataset, http://www.ifp.illinois.edu/~vuongle2/helen) (accessed on 1 May 2023), one can notice how the labeling process is meant to annotate the upper and lower lip contours, which do not necessarily result in clear mouth extremity landmarks. In addition, nose tipsn as well as the pupils’ centers, were absent among annotations.

Consequently, we enhanced the annotation of the training datasets with five additional well-defined landmarks: the two pupil centers, the nose tip, and the two mouth extremities. This process generated 17 fully defined facial landmarks, as illustrated in Figure 3.

### 4.2. Model Training

Our network was evaluated on standard benchmarks for facial landmark estimation, namely the 300w dataset presented by [44], HELEN by [45], and the WLFW by [46]. The model was trained from scratch without any transfer learning procedures and with a learning rate of $10^{-4}$, which was reduced to $10^{-5}$ in the 25th epoch and further reduced to $10^{-6}$ in the 40th epoch. We used a batch size of 16 images and adopted the Adam algorithm from [47] as the optimizer, with $\beta_{1}=0.9$ and $\beta_{2}=0.999$. The ground-truth heatmaps were created by inserting a 3×3 Gaussian kernel around the annotated joint coordinates. For comparison purposes, LandmarkDetector and then the LandmarkDetector–SpatialModel combination were trained for 120 epochs separately. The stopping criterion for the training process was 10 successive epochs without any enhancement of the training loss. Our data augmentation protocol consisted of an affine transform to each image of the current input batch. A uniform distribution was adopted to randomly set a scaling parameter between [1.05,1.5], a translation parameter between 0% and 10% of the image’s width and height, and a rotation parameter between [−20,20] degrees. Horizontal flipping was applied, with a probability of 0.5 at each image individually.

### 4.3. Model Evaluation

The model performance is first investigated qualitatively in Section 4.3.1. Thereafter, we propose our quantitative evaluation of the model’s performance in Section 4.3.2.

#### 4.3.1. Effect of Filtering on LandmarkDetector

**Figure 5:**
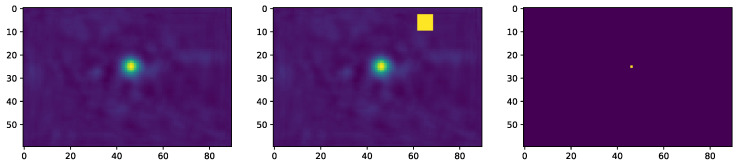
(**a**) LandmarkDetector’s output, (**b**) outlier introduction, (**c**) SpatialModel’s output (best viewed in color).

To better illustrate the impact of the spatial model mechanism on the initial heatmap estimations, we introduced a strong false positive signal by adding a stain located some pixels away and having the same intensity as the absolute maximum (the estimated landmark’s location) of the heatmap, as presented in Figure 5: One could notice that the spatial model SpatialModel not only suppressed the introduced outlier, but also generated a less blurry and less dilated blob around the landmark’s location. Such filtered signals should enhance the training performance once introduced to the backpropagation algorithm for LandmarkDetector training.

#### 4.3.2. Quantitative Evaluation

For accuracy evaluation, and based on the percentage of correct keypoints (PCK), defined by [48], and inspired by its adaptation, as in [49], we denoted by PCKp the PCK relative to the distance between two pupils’ centers: the left pupil center (LPc) and right pupil center (RPc) landmarks. We defined the threshold for the estimation success of a landmark as 10% of the current interpupillary distance.

The PCK for landmark *i* at frame *t* is calculated as follows:

(8) $${\mathrm{PCKp}}_{i}\left(r\right)=\frac{100}{N}\sum_{t=1}^{N}{∥x_{i}^{t*}-x_{i}^{t}∥}^{2}\leq 0.1r_{t}^{2}$$

where $r_{t}=∥x_{lpc}^{t}-x_{rpc}^{t}∥$ and $x_{i}^{t*}$, $x_{i}^{t}$ are the estimated and the ground-truth landmarks locations, respectively, and *N* is the number of the adopted frames.

In Table 1, we present the PCKp metric for five different landmarks, namely the left pupil center LPc; right pupil center, RPc; nose tip, NT; left mouth extremity, LMe; and right mouth extremity, RMe, for the above-mentioned datasets.

Similarly to PCK, we calculated the median of the normalized mean error (NME), which is normalized by the interpupillary distance, performed for the 300 w and WFLW datasets, and presented on the second row in Table 2. Even though our model’s performance outperformed some approaches, we were slightly under the state-of-the-art accuracy reported by [50] by 2.96%.

**Figure 6:**
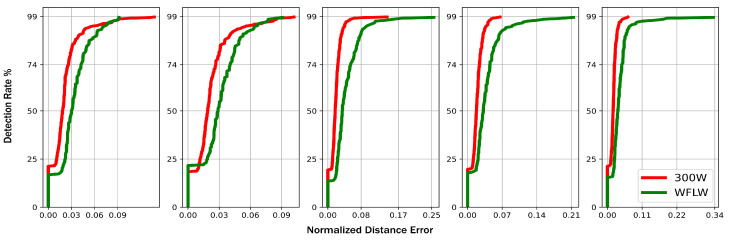
The cumulative point-to-point error distribution normalized by the interpupillary distance for LP center RP center, NT, LM extremity, and RM extremity, respectively (best viewed in color).

In order to allow a more exhaustive investigation of our model’s performance, in Figure 6, we present the cumulative point-to-point error distribution normalized by the interpupillary distance for the five above-mentioned landmarks for the 300 w and WFLW datasets. By comparing Figure 6 to the reported cumulative distributions by the references mentioned in Table 2, we can again identify the effect of the SpatialModel in dealing with false positives. In fact, for the 300 w dataset, we can spot that on average, 90% of the detected landmarks fall within 4% error. By extracting this same parameter, whenever available, from every reference in Table 2, we can report that we overcame all of them, as summarized in the third column from Table 2, and we achieved state-of-the-art performance for this parameter. Accordingly, we can again affirm that the postprocessing procedure helped to decrease the false positive occurrence.

## 5. Conclusions

In this work, it was demonstrated that integrating conditional probabilities from training data into a spatial model significantly improves the performance of the convolutional facial landmark detector. The consistency check performed by this spatial model enabled the training of the CNN on only 17 landmarks, leading to a lightweight model. Due to the carefully selected landmarks specifically focusing on the pupil region, this model allows for pupil region extraction for a further pupil size estimation from an unconstrained head pose. Training and inference of our model use ordinary hardware at almost real-time fps, allowing for its use in real-time pupil size estimation.

## Figures and Tables

**Table 1:** Percentage of correct keypoints, PCKp, for the 5 main landmarks for different datasets.

	LPc	RPc	NT	LMe	RMe
300 w	98.1	99.03	94.3	97.1	97.6
HELEN	99.0	99.4	98.4	96.4	98.7
WLFW	95.31	96.5	93.3	92.8	91.6

**Table 2:** Median of NME for (300w+WFLW) datasets and NME for the first 90% of (300 w).

Method	NME (300 w, WFLW)	NME < 90% (300 w)
LAB [46]	5.8, 5.27	6.5
MERGET [8]	5.29 (IBUG)	4.5, 7 (IBUG)
DVLN [51]	4.45, -	5.5
MTAAE [52]	4.3, 5.18	-
ODN [53]	3.56, -	9
HG-HSLE [54]	3.28, -	4.7
PIPNET [55]	3.19, 4.31	-
CE-CLM [56]	3.15, -	4.5
DTLD [50]	2.96, 4.05	-
OURS	3.3, 4.1	4.0

## Data Availability

All training data are open-source. References are indicated.

## References

[1] Lorenz B., Strohmayr E., Zahn S., Friedburg C., Kramer M., Preising M., Stieger K. (2012). Chromatic pupillometry dissects function of the three different light-sensitive retinal cell populations in RPE65 deficiency. Investig. Ophthalmol. Vis. Sci. IOVS.

[2] Takács B., Wechsler H. (1997). Detection of faces and facial landmarks using iconic filter banks. Pattern Recognit..

[3] Cootes T.F., Edwards G.J., Taylor C.J. (2001). Active appearance models. IEEE Trans. Pattern Anal. Mach. Intell..

[4] Kopaczka M., Acar K., Merhof D. Robust Facial Landmark Detection and Face Tracking in Thermal Infrared Images using Active Appearance Models. Proceedings of the VISIGRAPP.

[5] Cootes T.F., Taylor C.J., Cooper D.H., Graham J. (1995). Active shape models-their training and application. Comput. Vis. Image Underst..

[6] Hsu T.C., Huang Y.S., Cheng F.H. A novel ASM-based two-stage facial landmark detection method. Proceedings of the Pacific-Rim Conference on Multimedia (PCM).

[7] Wu Y., Hassner T., Kim K., Medioni G., Natarajan P. (2017). Facial landmark detection with tweaked convolutional neural networks. IEEE Trans. Pattern Anal. Mach. Intell..

[8] Merget D., Rock M., Rigoll G. Robust facial landmark detection via a fully-convolutional local-global context network. Proceedings of the IEEE conference on computer vision and pattern recognition (CVPR).

[9] Khan K., Attique M., Khan R.U., Syed I., Chung T.S. (2020). A multi-task framework for facial attributes classification through end-to-end face parsing and deep convolutional neural networks. Sensors.

[10] Deng J., Liu Q., Yang J., Tao D. (2016). M3 csr: Multi-view, multi-scale and multi-component cascade shape regression. Image Vis. Comput..

[11] Liu Q., Yang J., Deng J., Zhang K. (2017). Robust facial landmark tracking via cascade regression. Pattern Recognit..

[12] Xu C., Liao M., Li P., Guo Y., Liu Z. (2021). Bifurcation properties for fractional order delayed BAM neural networks. Cogn. Comput..

[13] Xu C., Mu D., Liu Z., Pang Y., Liao M., Li P., Yao L., Qin Q. (2022). Comparative exploration on bifurcation behavior for integer-order and fractional-order delayed BAM neural networks. Nonlinear Anal. Model. Control.

[14] Xu C., Zhang W., Aouiti C., Liu Z., Yao L. (2023). Bifurcation insight for a fractional-order stage-structured predator–prey system incorporating mixed time delays. Math. Methods Appl. Sci..

[15] Xu C., Liu Z., Li P., Yan J., Yao L. (2022). Bifurcation Mechanism for Fractional-Order Three-Triangle Multi-delayed Neural Networks. Neural Process Lett..

[16] Xu C., Mu D., Liu Z., Pang Y., Liao M., Aouiti C. (2023). New insight into bifurcation of fractional-order 4D neural networks incorporating two different time delays. Commun. Nonlinear Sci. Numer. Simul..

[17] Medley D.O., Santiago C., Nascimento J.C. (2019). Deep active shape model for robust object fitting. IEEE Trans. Image Process..

[18] Moldovanu S., Toporaș L.P., Biswas A., Moraru L. (2020). Combining sparse and dense features to improve multi-modal registration for brain DTI images. Entropy.

[19] Chen L., Su H., Ji Q. (2019). Deep structured prediction for facial landmark detection. Adv. Neural Inf. Process. Syst..

[20] Tompson J.J., Jain A., LeCun Y., Bregler C. (2014). Joint training of a convolutional network and a graphical model for human pose estimation. Adv. Neural Inf. Process. Syst..

[21] Yue-Hei Ng J., Yang F., Davis L.S. Exploiting local features from deep networks for image retrieval. Proceedings of the IEEE Conference on Computer Vision and Pattern Recognition Workshops.

[22] Sun Y., Wang X., Tang X. Deep convolutional network cascade for facial point detection. Proceedings of the IEEE Conference on Computer Vision and Pattern Recognition (CVPR).

[23] Chen X., Zhou E., Mo Y., Liu J., Cao Z. Delving deep into coarse-to-fine framework for facial landmark localization. Proceedings of the IEEE Conference on Computer Vision and Pattern Recognition Workshops.

[24] Zhang Z., Luo P., Loy C.C., Tang X. Facial landmark detection by deep multi-task learning. Proceedings of the European Conference on Computer Vision (ECCV).

[25] He Z., Kan M., Zhang J., Chen X., Shan S. A fully end-to-end cascaded cnn for facial landmark detection. Proceedings of the 2017 12th IEEE International Conference on Automatic Face & Gesture Recognition (FG).

[26] Gogić I., Ahlberg J., Pandžić I.S. (2021). Regression-based methods for face alignment: A survey. IEEE Signal Process. Mag..

[27] Springenberg J.T., Dosovitskiy A., Brox T., Riedmiller M. (2014). Striving for simplicity: The all convolutional net. arXiv.

[28] Hannane R., Elboushaki A., Afdel K. (2020). A divide-and-conquer strategy for facial landmark detection using dual-task CNN architecture. Pattern Recognit..

[29] Long J., Shelhamer E., Darrell T. Fully convolutional networks for semantic segmentation. Proceedings of the IEEE Conference on Computer Vision and Pattern Recognition (CVPR).

[30] Ronneberger O., Fischer P., Brox T. U-net: Convolutional networks for biomedical image segmentation. Proceedings of the International Conference on Medical image computing and computer-assisted intervention (MICCAI).

[31] Newell A., Yang K., Deng J. Stacked hourglass networks for human pose estimation. Proceedings of the European Conference on Computer Vision (ECCV).

[32] Bulat A., Tzimiropoulos G. Human pose estimation via convolutional part heatmap regression. Proceedings of the European Conference on Computer Vision (ECCV).

[33] Erhan D., Courville A., Bengio Y., Vincent P. Why does unsupervised pre-training help deep learning?. Proceedings of the Thirteenth International Conference on Artificial Intelligence and Statistics (AISTATS)—JMLR Workshop and Conference Proceedings.

[34] Ren J., Chen X., Liu J., Sun W., Pang J., Yan Q., Tai Y.W., Xu L. Accurate single stage detector using recurrent rolling convolution. Proceedings of the IEEE Conference on Computer Vision and Pattern Recognition (CVPR).

[35] Van Noord N., Postma E. (2017). Learning scale-variant and scale-invariant features for deep image classification. Pattern Recognit..

[36] Xu Y., Xiao T., Zhang J., Yang K., Zhang Z. (2014). Scale-invariant convolutional neural networks. arXiv.

[37] Kim S.W., Kook H.K., Sun J.Y., Kang M.C., Ko S.J. Parallel feature pyramid network for object detection. Proceedings of the European Conference on Computer Vision (ECCV).

[38] Jain A., Tompson J., Andriluka M., Taylor G.W., Bregler C. Learning human pose estimation features with convolutional networks. Proceedings of the International Conference on Learning Representations (ICLR).

[39] Moraru L., Moldovanu S., Dimitrievici L.T., Dey N., Ashour A.S., Shi F., Fong S.J., Khan S., Biswas A. (2019). Gaussian mixture model for texture characterization with application to brain DTI images. J. Adv. Res..

[40] Felzenszwalb P.F., Huttenlocher D.P. (2006). Efficient belief propagation for early vision. Int. J. Comput. Vis..

[41] Wang X., Bo L., Fuxin L. Adaptive wing loss for robust face alignment via heatmap regression. Proceedings of the IEEE/CVF International Conference on Computer Vision (ICCV).

[42] Seshadri K., Savvides M. Robust modified active shape model for automatic facial landmark annotation of frontal faces. Proceedings of the 2009 IEEE 3rd International Conference on Biometrics: Theory, Applications, and Systems (BTAS).

[43] Milborrow S., Nicolls F. Locating facial features with an extended active shape model. Proceedings of the European Conference on Computer Vision (ECCV).

[44] Sagonas C., Antonakos E., Tzimiropoulos G., Zafeiriou S., Pantic M. (2016). 300 faces in-the-wild challenge: Database and results. Image Vis. Comput..

[45] Le V., Brandt J., Lin Z., Bourdev L., Huang T.S. Interactive facial feature localization. Proceedings of the European Conference on Computer Vision (ECCV).

[46] Wu W., Qian C., Yang S., Wang Q., Cai Y., Zhou Q. Look at boundary: A boundary-aware face alignment algorithm. Proceedings of the IEEE Conference on Computer Vision and Pattern Recognition (CVPR).

[47] Kingma D.P., Ba J. (2014). Adam: A method for stochastic optimization. arXiv.

[48] Yang Y., Ramanan D. (2012). Articulated human detection with flexible mixtures of parts. IEEE Trans. Pattern Anal. Mach. Intell..

[49] Andriluka M., Pishchulin L., Gehler P., Schiele B. 2d human pose estimation: New benchmark and state of the art analysis. Proceedings of the IEEE Conference on Computer Vision and Pattern Recognition (CVPR).

[50] Li H., Guo Z., Rhee S.M., Han S., Han J.J. Towards Accurate Facial Landmark Detection via Cascaded Transformers. Proceedings of the IEEE/CVF Conference on Computer Vision and Pattern Recognition (CVPR).

[51] Wu W., Yang S. Leveraging intra and inter-dataset variations for robust face alignment. Proceedings of the IEEE Conference on Computer Vision and Pattern Recognition Workshops.

[52] Yue X., Li J., Wu J., Chang J., Wan J., Ma J. (2021). Multi-task adversarial autoencoder network for face alignment in the wild. Neurocomputing.

[53] Zhu M., Shi D., Zheng M., Sadiq M. Robust facial landmark detection via occlusion-adaptive deep networks. Proceedings of the IEEE/CVF Conference on Computer Vision and Pattern Recognition (CVPR).

[54] Zou X., Zhong S., Yan L., Zhao X., Zhou J., Wu Y. Learning robust facial landmark detection via hierarchical structured ensemble. Proceedings of the IEEE/CVF International Conference on Computer Vision (ICCV).

[55] Jin H., Liao S., Shao L. (2021). Pixel-in-pixel net: Towards efficient facial landmark detection in the wild. Int. J. Comput. Vis..

[56] Zadeh A., Chong Lim Y., Baltrusaitis T., Morency L.P. Convolutional experts constrained local model for 3d facial landmark detection. Proceedings of the IEEE International Conference on Computer Vision Workshops.

